# *Plasmodium falciparum *genotypes diversity in symptomatic malaria of children living in an urban and a rural setting in Burkina Faso

**DOI:** 10.1186/1475-2875-8-135

**Published:** 2009-06-20

**Authors:** Issiaka Soulama, Issa Nébié, Alphonse Ouédraogo, Adama Gansane, Amidou Diarra, Alfred B Tiono, Edith C Bougouma, Amadou T Konaté, Gustave B Kabré, Walter RJ Taylor, Sodiomon B Sirima

**Affiliations:** 1 Centre National de Recherche et de Formation sur le Paludisme. 01 BP 2208 Ouagadougou 01, Burkina Faso; 2 Travel and Migration Medicine Unit, Geneva University Hospital, Geneva, Switzerland; 3 Oxford University Clinical Research Unit, Hanoi, Vietnam; 4 Groupe de Recherche et d'Action en Santé, Ouagadougou, Burkina Faso; 5 Université de Ouagadougou, Unité de Formation et de Recherche Science de la Vie et de la Terre, Ouagadougou, Burkina Faso

## Abstract

**Background:**

The clinical presentation of malaria, considered as the result of a complex interaction between parasite and human genetics, is described to be different between rural and urban areas. The analysis of the *Plasmodium falciparum *genetic diversity in children with uncomplicated malaria, living in these two different areas, may help to understand the effect of urbanization on the distribution of *P. falciparum *genotypes.

**Methods:**

Isolates collected from 75 and 89 children with uncomplicated malaria infection living in a rural and an urban area of Burkina Faso, respectively, were analysed by a nested PCR amplification of *msp1 *and *msp2 *genes to compare *P. falciparum *diversity.

**Results:**

The K1 allelic family was widespread in children living in the two sites, compared to other *msp1 *allelic families (frequency >90%). The MAD 20 allelic family of *msp1 *was more prevalent (*p *= 0.0001) in the urban (85.3%) than the rural area (63.2%). In the urban area, the 3D7 alleles of *msp2 *were more prevalent compared to FC27 alleles, with a high frequency for the 3D7 300_bp _allele (>30%). The multiplicity of infection was in the range of one to six in the urban area and of one to seven in the rural area. There was no difference in the frequency of multiple infections (p = 0.6): 96.0% (95% C.I: 91.6–100) in urban versus 93.1% (95%C.I: 87.6–98.6) in rural areas. The complexity of infection increased with age [p = 0.04 (rural area), p = 0.06 (urban area)].

**Conclusion:**

Urban-rural area differences were observed in some allelic families (MAD20, FC27, 3D7), suggesting a probable impact of urbanization on genetic variability of *P. falciparum*. This should be taken into account in the implementation of malaria control measures.

## Background

According to demographic [1] and entomological [2] data, urbanization itself can reduce malaria transmission and increase the number of non-immune individuals, leading to differences in the features of rural and urban malaria. Previous epidemiological studies carried out in malaria-endemic areas highlighted the predominance of cerebral symptoms of severe malaria in low transmission urban area, while in high transmission rural areaa severe anaemia was the main clinical feature of severe malaria [3].

The relationship between parasites genotypes and clinical outcome has been reported in several studies [4-6]. The influence of the urbanization on transmission level and consequently on the clinical outcome, may affect the allelic polymorphism of *Plasmodium falciparum *and the distribution of parasite genotypes between urban and rural areas. A better understanding of the rural-urban dynamics of *P. falciparum *genotypes may be an important element for implementing malaria control strategies in the sub-Saharan Africa.

The present study examined and compared the genetic diversity of *P. falciparum *in infected children living in two different areas, rural and urban, in Burkina Faso using two highly polymorphic genes encoding the merozoite surface protein-1 (MSP-1) and the merozoite surface protein-2 (MSP-2) [7,8].

## Methods

### Study sites

The study was carried out in 2003 in the Health district of Pissy in Ouagadougou, the capital of Burkina Faso, and in Balonghin, a village in the Saponé Health district. The Pissy Health district is one of the four urban Health districts of the Ouagadougou city located at the west part of the city. The Pissy Health district catchments area is inhabited by about 638,234 inhabitants. According to the National Statistic and Demography Institute report, the literacy rate in Ouagadougou city was about 63% in 2003 [9]. The Saponé Health district is located in the centre-south region at about 50 kilometres south of Ouagadougou. This district is one of fifty rural districts of the country. The population size of Saponé health district was estimated at 105,157 inhabitants with a regional literacy rate at about 15.9% [9].

In the Balonghin rural area, houses are mainly constructed out of local materials with mud block and roofed with the same material. Some mud block houses were roofed with iron-sheet as well as grass. The Pissy urban area was mainly characterized by housing unit built with manufactured material (cement) and roofed with iron-sheet.

Malaria transmission is perennial in both districts with a peak during the rains season (from May to October). The entomological inoculation rate (EIR) was estimated at < 10 infected bites/person/year in Ouagadougou [10] and 200 infected/bites/person/year in Balonghin area [11]. In 2003, the prevalence rate in children of less than five years of age, presenting with clinical malaria, was estimated at 37.6% and 43.9%, in Pissy and Saponé health district, respectively [12].

### Patient enrolment

The study was part of studies assessing anti-malarial drug efficacy that were performed from July to December 2003 at both sites. Patients were enrolled in accordance with the World Health Organization (WHO) protocol for anti-malarial assessment [13], but slightly modified. The inclusion criteria included: 1) age between six and 59 months; 2) fever (axillary temperature = 37.5°C) or a history of fever within the past 48 hours; 3) *P. falciparum *mono-infection with parasite density between 1,000 and 150,000 asexual forms per microlitre, identified microscopically on blood smears; 4) no history of anti-malarial drug administration in the last two weeks; 5) no history of serious adverse effects to the study drugs (chloroquine and sulphadoxine-pyrimethamine); 6) no evidence of a concomitant febrile illness; 7) no sign/symptoms of severe malaria as defined by WHO [14].

Informed consent was obtained from parents or legal guardians of children prior to their enrolment. At the time of the study, there was no Ethical Committee in Burkina Faso; therefore, the study protocol was reviewed and approved by The Centre National de Recherche et de Formation sur le Paludisme of the Ministry of Health of Burkina Faso.

Blood spots were collected on filter paper from all children fulfilling the above criteria. Thick and thin blood films were prepared and read in the standard way; the number of parasites per microlitre of blood was calculated assuming an average of 8,000 white blood cell/μl of blood. For the internal quality control, 10% of all slides were re-read by an experienced independent microscopist.

### Blood spots samples and parasite DNA extraction

Blood from a finger prick spotted onto Whatman filter Paper #1, labelled with patients' study numbers, air-dried, and individually placed into plastic bag marked with the patients' initials. The bags were stored at room temperature. Parasite DNA was extracted using Chelex methods [15]. Briefly, 50 μl of 20% Chelex 100 solution (Bio-Rad Laboratories) was added to 1.5 ml microcentrifuge tube containing fragments of filter paper sample. Then, 100 μl of sterile water were added and the microcentrifuge tube placed onto a heating block at 95–100°C for 10 minutes of incubation. During the incubation phase, the tube was gently whirled and returned to the heat block every two minutes. The samples were centrifuged twice and the final supernatant was conserved in a new, labelled tube and stored at -20°C until it was used for the amplification reaction.

### PCR amplification of the *P. falciparum msp1 *and *msp2 *genes

Two polymorphic loci, *msp1 *and *msp2*, were used for the genotyping of the parasite population in this study. The regions of *msp1 *and *msp2*, which vary in repeat number and in adjacent sequence type, with three (Mad20, K1, RO33) and two (FC27, 3D7) allelic families [16], were analysed by a nested PCR amplification. For the primary amplifications, outer primer pairs corresponding to the flanking sequence of the conserved regions of *msp1 *and *msp2 *were used. The second amplification reactions were based on the primary products using allelic-specific primers sets corresponding to K1, RO33 and Mad20 families of *msp1 *and FC27, 3D7 families of *msp2*.

The following oligonucleotide primers sequences were used:

*msp2-1 *outer (ATG AAG GTA ATT AAA ACA TTG TCT ATT ATA); *msp2-4 *outer (ATA TGG CAA AAG ATA AAA CAA GTG TTG CTG); *msp1-A *outer (AAG CTT TAG AAG ATG CAG TAT TGA C); *msp1-B *outer (ATT CAT TAA TTT CTT CAT ATC CAT C); *FC27-A *(GCA AAT GAA GGT TCT AAT ACT AAT AG); *FC27-B *(GCT TTG GGT CCT TCT TCA GTT GAT TC); *3D7-A *(GCA GAA AGT AAG CCT TCT ACT GGT GCT); *3D7-B *(GAT TTG TTT CGG CAT TAT TAT GA); *K1-A *(AAG AAA TTA CTA CAA AAG GTG CAA GTG); *K1-B *(AGA TGA AGT ATT TGA ACG AGG TAA AGT G); *RO33-A *(AGG ATT TGC AGC ACC TGG AGA TCT); *RO33-B *(GAG CAA ATA CTC AAG TTG TTG CAA AGC); *MA20-A *(TGA ATT ATC TGA AGG ATT TGT ACG TCT TGA); *MAD 20-B *(GAA CAA GTC GAA CAG CTG TTA)

Primary amplifications reactions were carried out using 5 μl of DNA extracted solution and the nested PCR used 2 μl of the first PCR product. For each reaction, 200 μM each of dNTP, 1 μM of each primer, 0.5 UI of *Taq DNA polymerase *(Sigma Aldrich Chemie GMBH, Deutshland) and PCR Buffer 10× containing 100 mM Tris-HCl, pH 8.3 at 25°C;500 mM KCl; 15 mM MgCl2; and 0.01% gelatine, were used in a final volume of 20 μl. First and nested amplifications were performed on a PELTIER PTC Thermocycler 100. The cycle condition for msp1 and msp2 outer PCR (30 cycles) were: denaturation at 94°C for 5 min, annealing at 55°C for 1 min 30 sec, extension at 72°C for 2 min and final extension for 10 min to insure that all product were full-length. For the inner PCR similar conditions were used for the denaturing and extension but the annealing condition were specific for each primer as described: FC27 and 3D7 were annealing at 57°C, K1 and MAD20 were annealing at 62°C, RO33 annealing temperature was 58°C. The specific amplifications in *msp1 *group were performed for 25 cycles while for *msp2 *group the reaction were 30 cycles.

The amplified PCR products were either stored at +4°C or analysed immediately by electrophoresis on a 1.5% agarose gel (Sigma Aldrich Chemie GMBH, Deutshland). The sizes of the PCR products were estimated using 100 base pairs (bp) DNA ladder marker (Sigma Aldrich Chemie GMBH). The *msp1 *and *msp2 *alleles were categorized by their molecular weights and considered the same if their molecular weights were approximately within 10 bp.

### Definitions

The detection of a single PCR fragment for each locus was classified as an infection with one parasite genotype. The detection of more than one PCR fragment for either *msp1 *or *msp2 *loci (i.e. an infection with more than one parasite genotype) defined as a multiple *P. falciparum *infection. The number of patients with more than one parasite genotype within the total infected population is defined as the frequency of multiple infections. The complexity was defined as the mean number of parasite genotypes per infected patient [17]. The complexity of infection and the frequency of multiple infections were calculated by combining the *msp1 *and *msp2 *PCR genotyping results. The highest number of bands detected, whatever the locus, was used to calculate the value for the overall complexity of infection.

### Statistical analysis

The DNA fragments from the electrophoresis were assigned to specific allelic families according to the second PCR results. The data were double entered and were analysed using Epi info Version 6.04. The proportions comparison was made by Chi squared and normally distributed continuous data by the Student's t test and ANOVA.

## Results

### Characteristics of the study population

A total of 164 filter blots samples were obtained; 75 and 89 from rural and urban dwellers, respectively. The mean age of the study participants from the two sites was similar (*p *= 0.3): 2.6 years [95% confidence interval (CI), 2.3–2.9] and 2.4 years [95% CI 1.4–3.4] in rural and urban area, respectively. The male to female ratios were 1.1 and 1.2 in Balonghin (rural) and Pissy (urban), respectively.

The nested PCR of *msp1 *and *msp2 *amplification were completed on all samples from the two groups. *Plasmodium falciparum *DNA amplification was successful for *msp1 *and *msp2 *in all 75 (100%) samples from Pissy, but of 89 samples from Balonghin, 87 (97.8%) and 73 (82.0%) samples were successfully amplified for *msp1 *and for *msp2*, respectively.

### Complexity and frequency of multiple infections

**Figure 1:**
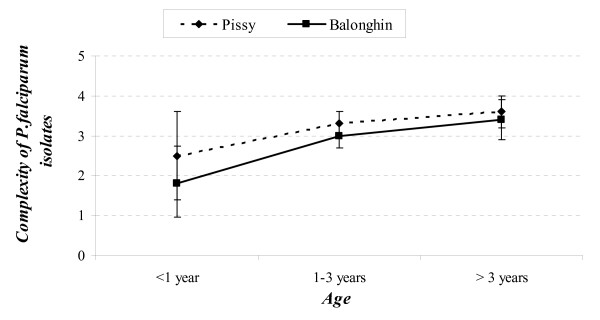
**Mean number (complexity) of *Plasmodium falciparum *genotypes among children from Balonghin (rural area) and Pissy (urban area)**. *p-value (t-test in rural area) = 0.04; p-value (t-test in urban area) = 0.06*.

The numbers of different parasite genotypes found in infected children were ranged from one to seven in the rural area and from one to six in the urban area. The complexity of infection (Figure 1) did not show any statistical difference between the rural and urban areas (p = 0.1). However, there was a trend of increasing complexity with age in the rural area with a positive association (*p *= 0.04, t-test), but with a borderline statistical significance (*p *= 0.06, t-test) in the urban area (Figure 1). Based on the two genetic loci, the frequency of multiple infections was >90% in all children and there was no significant difference (p = 0.3) between rural 92.0 (86.3–97.7)% and urban 96.0 (92.0–100)% areas.

### Prevalence of *msp1 *and *msp2 *allelic families

Of all the alleles analysed, the K1-type alleles were the most frequently detected in symptomatic malaria children living in both areas (Additional file 1). Overall, the frequencies of the K1 and RO33 type alleles showed no difference between symptomatic malaria children in the two areas while the Mad20-type alleles were significantly more frequent in the urban area malaria children. Of the *msp2 *alleles, the FC27 type alleles were more frequent in the rural symptomatic children, compared to those living in the urban area, and occurred at a higher frequency than the 3D7 type allele in the rural area. The 3D7 type alleles were more frequent in symptomatic malaria cases in the urban area compared to those from rural area (not statistically significant), but significantly more prevalent than the FC27 allele in the urban area.

### Distribution of *msp1 *and *msp2 *alleles

**Figure 2:**
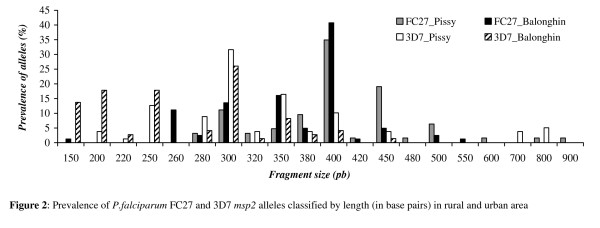
**Prevalence of *P. falciparum *FC27 and 3D7 *msp2 *alleles classified by length (in base pairs) in rural and urban area**.

**Figure 3:**
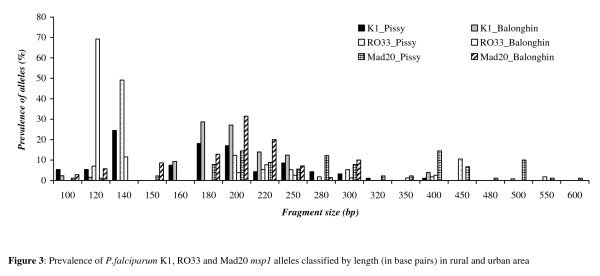
**Prevalence of *P. falciparum *K1, RO33 and Mad20 *msp1 *alleles classified by length (in base pairs) in rural and urban area**.

All the *msp1 *and *msp2 *alleles were classified according to their size. Seventy individual *msp *alleles were identified in study children in the two sites (Figures 2 and 3): 16 different alleles for FC27 (150–900 bp); 13 for 3D7 (150–800 bp); 13 K1 type's alleles (100–500 bp); 11 alleles for RO33 (120–550 bp) and 17 Mad20 types alleles (100–600 bp).

The sizes of all the FC27 type alleles from children living in the urban area were between 280 and 900 bp while in rural area there were few children with alleles above 550 bp (Figure 2); overall, fragments of the 400 bp allele predominated in children from the both areas: 35% in infected children from the urban and 40% in children from the rural area. The FC27 350_bp_allele was more prevalent in malaria infected children from rural than those from the urban area (*p *= 0.02) while FC27 450_bp _was more prevalent in urban symptomatic children than in rural children *(p = 0.02)*. The most prevalent infecting 3D7 allele was the 3D7 300 _bp _in both areas [31.6% in urban and 26% in rural area (Figure 2)].

The analysis of K1 alleles revealed four prevalent alleles (180_bp_, 200_bp_, 220_bp _and 250_bp_) in the rural malaria infected patients versus two in the urban malaria infected patients (200 and 220_bp_). The K1 140_bp _allele was absent in children infected in rural area (Figure 3).

Of the RO33 alleles, the RO33_120 bp _(69%) and RO33 140_bp _(49%) were dominant in malaria-infected children in rural and urban areas, respectively. The Mad20 type alleles were more frequent in urban malaria infected children (17 different alleles) compared to rural area (nine different alleles) (Figure 3).

## Discussion

The purpose of this study was to compare, using the two most polymorphic regions of *msp1 *and *msp2 *genes, the genetic diversity of *P. falciparum *in malaria symptomatic children living in two different settings, an urban and a rural area, characterized by markedly different transmission intensities.

Although all the *msp1 *families (K1, Mad20 and RO33) were found in infected children from both areas, Mad 20 allelic family was found more frequent in infected children from urban area than those from rural area. But, some *msp1*alleles detected were differently distributed in children living in both sites. The K1 type alleles were the most representative's *msp1 *alleles in malaria infected children living both in the rural and the urban areas, consistent with different studies conducted in Gabon [18,19], in Central Africa or in Honduras [20] in Central America. However, a high frequency of the K1 140_bp _and RO33 140_pb _alleles was observed in children in urban site compared to a high frequency of RO33 120_pb _allele observed in those from rural area. These differences in urban rural symptomatic malaria children may suggest a specific immune response against this allele or a random event due to genetic drift [21].

The analysis of *msp2 *families showed the FC27 was predominant in children from the rural area whilst in the urban area most of the children was infected by the 3D7 family parasites When analysed the FC27 alleles, the FC27 350_pb _was predominant in children from the rural area compared to the FC27 450_pb _mainly observed in children infected the urban area. Differences in transmission level and anti-malarial immunity known to be strain specific [22], could partly explain the differences in the distribution of the different alleles. It was known that there was an increase of the migration process from the rural area to the urban area (Ouagadougou) the past 10 years [23]. This demographic change in the urban area, as well as others factors, such as individual and household factors (e.g. the use of impregnated bed nets, indoor insecticide spraying), and climatic and topographical factors (e.g. humidity, rain fall, temperature, soil type, the presence of water reservoirs) may affect the level of malaria transmission and lead to a rural-urban difference in parasite genotypes carriage.

In a given locality, the parasite genetic pool may be relatively stable, perhaps related to the stable parasite life cycle; thus, the distribution of alleles may be determined randomly so that certain alleles will predominate by chance. Nevertheless, there are certain factors external to the natural life cycle that are known to affect allelic distribution, notably, anti-malarial drug pressure that is characteristically greater in urban areas and result in a higher prevalence of drug resistance markers [24], the proximity of populations to water reservoirs, as it has been observed in Pissy [25], the use of insecticide-treated nets or certain human genetic factors (haemoglobin types, G6PD), that could explain the difference in the distribution of *P. falciparum *genotypes between the two sites.

The study did not show a difference in the complexity of infections between rural and urban area (3.0 ± 1.3 vs 3.3 ± 1.1 genotypes respectively) infected children less than five years of age. The data on *P. falciparum *complexity in children living in rural area were comparable to those observed in the eastern rural area of Burkina [26]. However, the data analysis also showed that the complexity of infection in both sites increased with children age; this was significant in rural and of borderline significance in urban area. This suggests that younger children might still have protection from maternal antibodies or could simply mean a lower risk of multiple infections due to a lower exposure time at risk of infection. A comparable trend of a positive association between complexity and age was observed in previous study in a rural area of Burkina Faso [26], but Issifou *et al *[27] did not observe an age-dependent pattern in Benin. Other factors, such malaria transmission intensity or transmission season may probably affect the variation in the complexity of infection [28-30].

## Conclusion

The paper demonstrated that there were some differences in the *P. falciparum *diversity between symptomatic children living in urban and rural areas and this should be taken into account when designing MSP1 or MSP2 malaria vaccine. The study also emphasizes the importance of evaluating the extent of parasite genetic variation and the factors affecting this variation. In this context, longitudinal studies examining the dynamics of the *P*. *falciparum *genetic diversity, including genes conferring drug resistance, between urban and rural areas, could have public health implications for malaria control.

## Competing interests

The authors declare that they have no competing interests.

## Authors' contributions

IS, IN and GK participated to study design, data collection, local supervision, statistical analysis, data interpretation and manuscript preparation. AO, AG, AD, AT, ECB, and AK participated to study design, data collection and local supervision. WT participated in revision of the manuscript. SS participated in the design of the study, acquisition of funding, statistical analysis data interpretation, coordination and writing of the manuscript.

## Supplementary Material

Additional file 1 **Table 1**. *msp1 *and *msp2 *families' distribution according to urban and rural area. Click here for file
