# Supplementary material for: Plasmodium falciparum genotypes diversity in symptomatic malaria of children living in an urban and a rural setting in Burkina Faso
Source: Malar J. 2009 Jun 20;8:135. doi: 10.1186/1475-2875-8-135 (PMC2705376; doi:10.1186/1475-2875-8-135)
Supplement: Additional file 1 — Table 1. msp1 and msp2 families' distribution according to urban and rural area. [file 1475-2875-8-135-S1.pdf]

Table 1: *msp1* and *msp2* families' distribution according to urban and rural area

Allelic family		Urban area (Pissy)			Rural area (Balonghin)			
Msp1		n	%	(95%CI)	n	%	(95 %CI)	*p*
	K1	70	93.3	(87.4-99.1)	83	95.4	(90.9-99.9)	*0.8*
	RO33	54	72.0	(60.0-83.9)	69	79.3	(69.7-88.9)	*0.3*
	Mad20	64	85.3	(76.6-94.0)	55	63.2	(50.5-75.9)	*0.001*
	p	*0.002*			*0.000001*			
	p(K1vs RO33)		*0.0006*			*0.0003*		
	p(K1 vs MAD20)		*0.11*			*<<0.00001*		
	p(RO33vs MAD20)		*0.05*			*0.02*		
Msp2								
	FC27	41	54.7	(36.5-69.9)	63	86.3	(77.8-94.8)	*0.00003*
	3D7	61	81.3	(71.5-91.1)	50	68.5	(55.6- 81.4)	*0.07*
	p	*0,0005*			*0,01*			
